# Vesicle condensation induced by synapsin: condensate size, geometry, and vesicle shape deformations

**DOI:** 10.1140/epje/s10189-023-00404-5

**Published:** 2024-01-25

**Authors:** Jette Alfken, Charlotte Neuhaus, András Major, Alyona Taskina, Christian Hoffmann, Marcelo Ganzella, Arsen Petrovic, David Zwicker, Rubén Fernández-Busnadiego, Reinhard Jahn, Dragomir Milovanovic, Tim Salditt

**Affiliations:** 1 Institut für Röntgenphysik, Georg-August-Universität, Friedrich-Hund-Platz 1, 37077 Göttingen, Germany; 2 Molekulare Neurowissenschaften, Deutsches Zentrum für Neurodegenerative Erkrankungen (DZNE), Charitéplatz 1, 10117 Berlin, Germany; 3 Labor für Neurobiologie, Max-Planck-Institut für multidisziplinäre Naturwissenschaften, Am Fassberg 11, 37077 Göttingen, Germany; 4 Institut für Neuropathologie, Universitätsmedizin Göttingen, Justus-von-Liebig-Weg 11, 37077 Göttingen, Germany; 5 Theorie Biologischer Flüssigkeiten, Max-Planck-Institut für Dynamik und Selbstorganisation, Am Fassberg 11, 37077 Göttingen, Germany

## Abstract

**Abstract:**

We study the formation of vesicle condensates induced by the protein synapsin, as a cell-free model system mimicking vesicle pool formation in the synapse. The system can be considered as an example of liquid–liquid phase separation (LLPS) in biomolecular fluids, where one phase is a complex fluid itself consisting of vesicles and a protein network. We address the pertinent question why the LLPS is self-limiting and stops at a certain size, i.e., why macroscopic phase separation is prevented. Using fluorescence light microscopy, we observe different morphologies of the condensates (aggregates) depending on the protein-to-lipid ratio. Cryogenic electron microscopy then allows us to resolve individual vesicle positions and shapes in a condensate and notably the size and geometry of adhesion zones between vesicles. We hypothesize that the membrane tension induced by already formed adhesion zones then in turn limits the capability of vesicles to bind additional vesicles, resulting in a finite condensate size. In a simple numerical toy model we show that this effect can be accounted for by redistribution of effective binding particles on the vesicle surface, accounting for the synapsin-induced adhesion zone.

**Graphic abstract:**

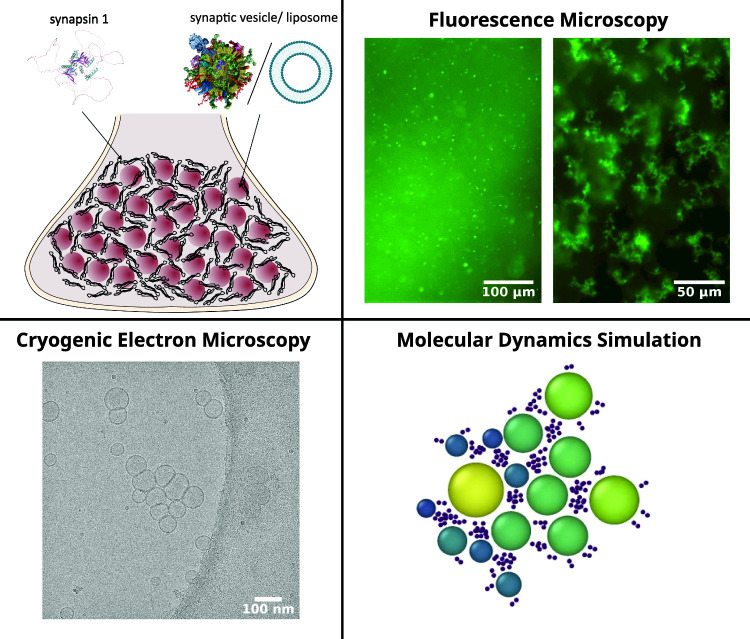

**Supplementary Information:**

The online version contains supplementary material available at 10.1140/epje/s10189-023-00404-5.

## Introduction

Liquid–liquid phase separation (LLPS) of biomolecular fluids has been proposed as a possible mechanism explaining the recent observations of mesoscale droplet phases in the cytosol of biological cells [16, 23, 29]. Such droplets can effectively form functional organelles, which are not bound by a membrane, and are currently under intensive investigation in cellular biology and biophysics. Interestingly, droplets form not only in many protein solutions, but can also be observed in mixtures of lipid vesicles and certain proteins, which induce coalescence into dense condensates induced by specific interactions between lipids and proteins [2, 18, 19]. In fact, vesicle protein interactions and deformation of condensates have been shown to play a vital role in many biological phenomena ranging from viral replication [10, 11] to vesiculary secretion [33].

A biologically particularly interesting and functionally relevant example of vesicular condensates are the tight clusters of synaptic vesicles (SVs) formed at the synapses, also denoted as a reserve vesicle pool providing SVs for neurotransmitter release during prolonged and repetitive rounds of neuronal stimulation. Neuronal communication and synaptic transmission is well known to rely on SVs which contain the neurotransmitters in the vesicle lumen and have a mean diameter of about $d\simeq 40$ nm [30]. The pools act as a reservoir for exocytosis and neurotransmitter release. Pool formation has been explained by liquid–liquid phase separation induced by the protein family of synapsins, most notably synapsin I [18, 19], which is the most abundant protein in the synaptic cytosol. Moreover, the adequate molar ratio of synapsin, SVs and other synaptic proteins such as $\alpha$-synuclein, plays a crucial role for the mesoscale organization of SV clusters [12, 24]. Accordingly, the condensation of vesicles is likely to be governed by equilibrium physicochemical effects, as opposed to, for example, active motor proteins. But even in equilibrium, a wide range of possible interactions could play a role, including osmotic pressure, ionic strength, steric interactions, or elasticity of a gel-like lipid-protein network. Important aspects of synaptic pool formation therefore still remain unclear. For example, one would like to know which forces determine the inter-vesicle distance, and which effect limits the overall size of a cluster? More generally, there is a lack of understanding why droplet sizes in biological cells are stabilized in size and do not continue to aggregate, while the interfacial tension of the two LLPS phases would typically drive a continued coalescence and ultimately macroscopic domain formation. Which ’finite-size’ effect can hence stabilize a mesoscale droplet?

In this work, we use cell-free and well-controlled model systems of vesicles and synapsin I (herein referred to as synapsin) to study pool formation in equilibrium buffers by fluorescence light microscopy (FLM) and cryogenic electron microscopy (cryo-EM). In an attempt to reduce complexity and to achieve a high purity of the preparations, we mainly study artificial lipid vesicles (LV), composed of four different lipids mimicking the membrane of a synaptic vesicle. Our goal is to identify relevant physicochemical principles limiting the cluster size. The main hypothesis of the work is that the interplay between attractive lipid-synapsin interaction and repulsive elastic interactions due to elastic strains in the interacting vesicles results in a mean equilibrium cluster size. Notably, we present evidence that the membrane tension induced by the adhesion zones, which a vesicle has already formed in a condensate, limits its ability to bind additional condensates. In a simple toy model, we show that ’depletion of binding capability’ modeled as a re-partitioning of bond-forming ’synapsin particles’ on the vesicle surface can indeed explain a finite condensate size. Finally, we want to explore, whether overall cluster geometry and vesicle shapes in the cluster are accessible by current FLM and cryo-EM, and whether structural changes are observed as a function of lipid composition, and protein-to-lipid (*P*/*L*) ratio. In this first step to address this issue, we find that the vesicle-synapsin condensates can undergo shape transformation from a rather spherical droplet morphology to a more fractal appearing aggregate, depending on the ratio.

**Fig. 1:**
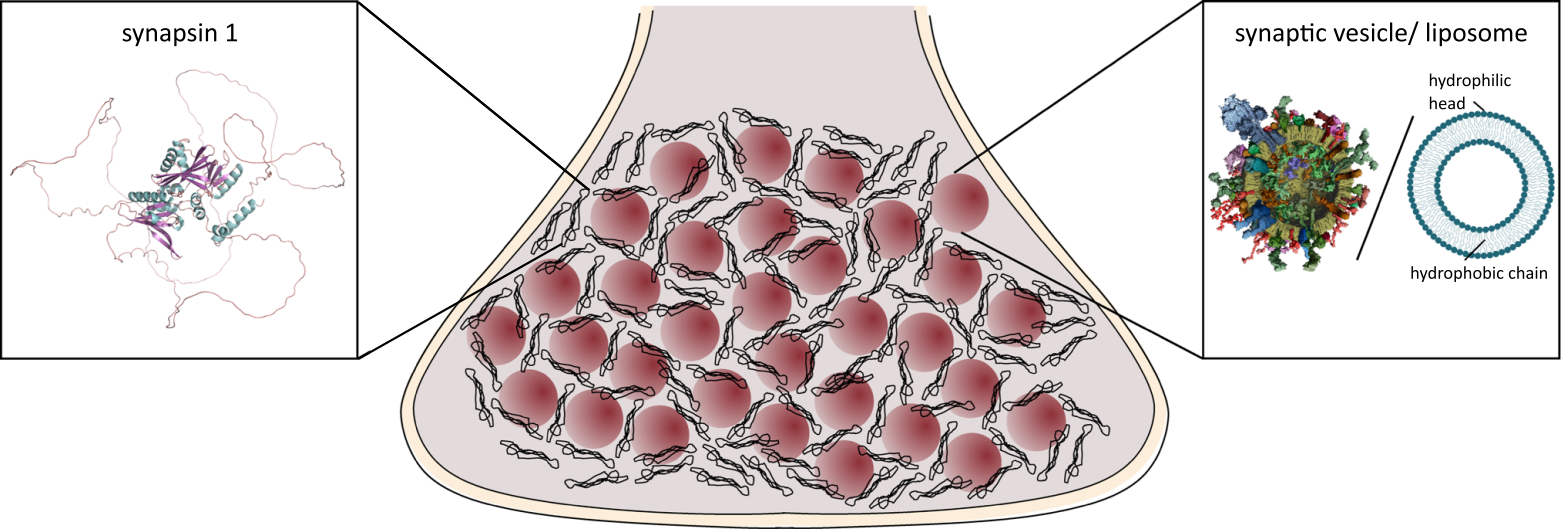
Vesicle pool model system consisting of synapsin and either synaptic vesicles (SV) or lipid vesicles (LV). The structure of synapsin I was predicted by AlphaFold and visualized with PyMOL [3, 26]. The SV illustration is taken from [30], the sketch of the artificial lipid vesicles illustrates the bilayer structure with hydrophilic headgroup and hydrophobic chains

Figure 1 shows a sketch of the model system consisting of synapsin I and synaptic vesicles or artificial lipid vesicles. Before turning to the vesicle-synapsin preparation protocol and the microscopy settings in the next Sect. 2, we want to give a brief account of what is known about synapsin, the ’effector’-protein of this work. Synapsin Ia has a length of 705 amino acids with a molecular weight of 74.11 kDa [32]. The structure of synapsin I predicted by AlphaFold and visualized with PyMOL is shown in Fig.  [3, 26]. Specific regions of synapsin I are referred to as intrinsically disordered regions (IDRs) as they do not fold into any stable secondary structure. Most of them are located in the C-terminal (residues 420–705) [27]. Synapsin is localized in larger amounts in the (pre)synapses of neurons as evidenced by immunostaining and fluorescent labeling [6], as well as quantitative mass spectrometry [36]. Furthermore, recent studies revealed a high concentration of synapsin at synaptic vesicles with a copy number of 8.3 copies/vesicle [30]. First investigations on the interaction of synapsin with vesicles disclosed that the head (N-terminal) of this protein binds to phospholipids and can interact with other membrane proteins [5]. It has also been demonstrated that both electrostatic and hydrophobic interactions contribute to the binding of synapsin to vesicles, e.g., for phosphatidylcholine (PC) lipids no binding occurred in contrast to phosphatidylserine (PS) lipids [8].

## Materials and methods

### Sample preparation

#### Synapsin I

EGFP-labeled synapsin I was expressed in Expi293 cells as described in [12, 19] and the purified protein was stored in a buffer consisting of 25 mM Tris–HCl (pH 7.4 at $4\,^{\circ }\textrm{C}$), 150 mM NaCl, and 0.5 mM TCEP. Following the purification, synapsin was snap-frozen in liquid nitrogen and stored at $-\,80\,^{\circ }\textrm{C}$ or on liquid nitrogen. Before the measurements, synapsin was thawed on ice.

#### Lipid vesicles (liposomes)

Lipid vesicles (LV) were prepared as follows: DOPC (1,2-di-(9Z-octadecenoyl)-sn-glycero-3-phosphocholine), DOPS (1,2-di-(9Z-octadecenoyl)-sn-glycero-3-phospho-L-serine), DOPE (1,2-di-(9Z-octadecenoyl)-sn-glycero- 3-phosphoethanolamine), and cholesterol were purchased from Avanti Polar Lipids (Alabaster, Al, USA) and Texas Red from fisher scientific (Hampton, USA). Lipids in powder form were then dissolved in chloroform and mixed in the desired concentrations. To mimic the charge distribution of synaptic vesicles, a mixture of 55 mol% DOPC, 20 mol% DOPS, 15 mol% DOPE and 10 mol% cholesterol was used. This 4 component system is also denoted as LV4. For control measurements, pure DOPC lipid vesicles without the anionic DOPS component were prepared. For fluorescence microscopy, 0.04 mol% TexadRed was added. Chloroform was evaporated using a stream of $\textrm{N}_2$ and a vacuum oven and the resulting lipid film was rehydrated in a buffer consisting of 25 mM Tris–HCl (pH 7.4 at $4\,^{\circ }\textrm{C}$), 150 mM NaCl and 0.5 mM TCEP. Vesicles were then formed by 10 freeze (liquid nitrogen) and thaw ($37\,^{\circ }\textrm{C}$ water bath) cycles followed by 21 extrusion cycles through a polycarbonate pore membrane, using the Avanti Polar Lipids Mini Extruder (Alabaster, Al, USA).

#### Synaptic vesicles

Synaptic vesicles (SVs) were extracted from rat brain following the protocol described in [30] and vesicles were resuspended in a sucrose buffer. After extraction, synaptic vesicles were snap-frozen and were kept frozen at $-\,80\,^{\circ }\textrm{C}$ or in liquid nitrogen and were thawed on ice for the measurements. SV suspensions prepared in this way have previously been characterized structurally by small-angle X-ray scattering (SAXS) [7, 15].

#### Preparation of synapsin-vesicle condensates

For condensate formation, synapsin I and vesicles were mixed on ice in the desired concentrations.

### Fluorescence microscopy

Fluorescence microscopy measurements were carried out using the Zeiss Axio Observer Z1 inverted microscope (Zeiss, Jena, Germany). EGFP-labeled synapsin I was excited at a wavelength of 470 nm and images were acquired with the monochromatic Axiocam Mrm CCD camera (Zeiss, Jena, Germany) with $1388 \times 1040$ pixels. The images were analyzed and processed using Fiji [25]. For the microscope recordings, the vesicle pool solution was either pipetted into $\mu -$slides ($\mu -\textrm{slide}$ 15 well 3D glass bottom, Ibidi, Gräfelfing, Germany) or in self-constructed sample chambers consisting of two glass coverslips connected by parafilm (Bemis, Wi, USA).

### Cryogenic electron microscopy

The vitrification of vesicles and synapsin was carried out with a Leica EM GP plunge-freezer (Leica Microsystems GmbH, Wetzlar, Germany). Quantifoil grids (Quantifoil R2/1, Quantifoil Micro Tools GmbH, Großlöbichen, Germany) were used for all experiments. The EM grids were treated with the plasma cleaner (Harrick Plasma, Ithaca, US) for 2 min before the sample application at ${10}\,^\circ \hbox {C}$ and a 90% relative humidity. The sample was then blotted for 5s (Leica Filter Paper Grade 595, $\varnothing$ 55/15 mm, Plano, Wetzlar, Germany) before plunge-freezing in a liquid ethane-propane bath (63% propane, 37% ethane) resulting in a thin layer of amorphous ice.

The cryo-EM experiments were performed with a Krios G4 microscope (Thermo Fisher Scientific) operating at 300 kV equipped with a field-emission gun and a Selectris X energy filter. The program SerialEM was used for data collection [17]. The exposure time was set to 2 s. The EM grids were tilted from − 60$^\circ$ to 60$^\circ$ with a step size of 3$^\circ$ for the acquisition of a tilt series. The images were analyzed and processed using Fiji [25]. A median filter with a size of 2 pixels was applied to all cryo-EM images.

### Molecular dynamics simulations

A simplified (toy) model of the condensate formation of lipid vesicles induced by synapsin was studied by Molecular dynamics (MD) simulation, carried out with the LAMMPS (Large-scale Atomic/Molecular Massively Parallel Simulator) package [31]. The simulations were performed in a 2D box with periodic boundary conditions and a size of $400 \times 400$ length units, with one unit corresponding to 10 nm. Lipid vesicles were described as polydisperse, round particles with radii sampled from a normal distribution with a mean of 4.17 (41.7 nm) and a standard deviation of 1.08 (10.8 nm). These parameters were chosen based on the vesicles size histograms measured by cryo-EM in the present work. Attractive interactions between particles were described by the cosine/square potential [9]

1 $$\begin{aligned} E(r) ={\left\{ \begin{array}{ll} -\varepsilon &{} r<\sigma ,\\ -\varepsilon \cos \left( \frac{\pi (r-\sigma )}{2(r_c-\sigma )}\right) ^2 &{} \sigma \le r <r_0\\ 0 &{} r\ge r_c \end{array}\right. } ~, \end{aligned}$$

with $\varepsilon$ denoting the depth of the potential well, $\sigma$ the equilibrium particle distance, and $r_c$ the interaction range. Repulsive interactions were modeled by the Weeks–Chandler–Anderson (WCA) potential [34]

2 $$\begin{aligned} E(r) = \varepsilon \left[ \left( \frac{\sigma }{r}\right) ^{12}-2\left( \frac{\sigma }{r}\right) ^6+1\right] ,\quad r<\sigma ~, \end{aligned}$$

with same parameter values for $\varepsilon$ and $\sigma$ as in the attractive cosine/square potential. The equilibrium distance between particles was set to $\sigma =2^{\frac{1}{6}}(r_1+r_2)$, and the interaction range to $r_c=\sigma +0.25$, where $r_1$ and $r_2$ denote the respective radii of the interacting particles. Synapsin-synapsin and vesicle-vesicle interactions were accounted for by the above attractive and repulsive potential according to the rules specified below. In addition, synapsins were tightly linked to a vesicle by a harmonic potential. They could rearrange on the vesicle surface, but not detach from their vesicle. Synapsin-synapsin interactions were described by attractive and repulsive potentials, depending on whether synapsins belonged to the same vesicle. The interactions between vesicles were chosen as purely repulsive and were hence only described by the WCA potential. Two different models for the description of synapsin were used.

#### Mono-domain synapsin model

In the first model, referred to as the mono-domain synapsin model, synapsin I was simulated as round particles with a radius of 0.5 (5 nm), corresponding to the radius of gyration of synapsin predicted by AlphaFold [3]. Each synapsin was linked to a vesicle by a bond with a harmonic potential, described by $E=\frac{1}{2}K(r-r_0)^2$ with the bond coefficient *K* set to $K=100$ and the equilibrium bond distance $r_0=2^{\frac{1}{6}}(r_1+r_2)$. Interactions between synapsins were considered to be attractive only when they belonged to different vesicles; otherwise, interactions were purely repulsive. This approach resembles the concept that longitudinal pairwise interactions are attractive, while lateral interactions are repulsive. Note that the repulsive lateral interaction in the simulation design serves to prevent aggregation of synapsins into one spot on the vesicle surface. It is not meant as a rule to specify the unknown issue of how exactly synapsin domains on the same or across two neighboring vesicles interact, in terms of sequence alignment, conformation and steric interaction.

#### Bi-domain synapsin model

In the second model, referred to as the bi-domain synapsin model, synapsin I was simulated as two adjacent round particles, each with a radius of 0.5. These two particles were treated as a rigid body, with fixed relative distance of 1. One round particle can be considered to represent the N-domain that interacts with the lipid vesicle, while the other represents the C-domain that interacts with the C-domains of other synapsins. Each N-domain was linked to a vesicle by a harmonic bond as previously described. Pairwise C-domain interactions were attractive, while N-domain interactions were purely repulsive. Generally, other interactions, such as C-N domains or vesicle-domain interactions, were purely repulsive.

For initialization, 200 vesicles were distributed in a box at random lattice nodes. Synapsin or the N-domains of synapsin, respectively, were uniformly positioned around the vesicle on a circle with a radius of $r=2^{\frac{1}{6}}(r_{\mathrm{{vesicle}}}+r_{\mathrm{{synapsin}}})$. In the bi-domain model, the centers of the synapsin N-domain and C-domain were aligned along the radial axis. The number of synapsin particles per vesicles was determined as

3 $$\begin{aligned} N= 2\pi ~ 2^{\frac{1}{6}} (r_{\mathrm{{vesicle}}}+r_{\mathrm{{synapsin}}})~\lambda , \end{aligned}$$

with the linear density $\lambda$. Pairwise interactions were only computed between particles within a neighbor list. Particles with a distance of $d\le 3.3$ were defined in the same neighbor list, the neighbor list was updated whenever an atom had moved by 0.15 or more. To model interaction with a background implicit solvent, Brownian dynamics were introduced through a Langevin Thermostat [31]

4 $$\begin{aligned} F=F_\textrm{c}+F_\textrm{f}+F_\textrm{r} ~, \end{aligned}$$

where $F_\textrm{c}$ describes the conservative force emerging from the attractive and repulsive interactions. A viscous damping force was accounted for by $F_\textrm{f}=-m v/\gamma$, and the collision of particles with solvent atoms by $F_\textrm{r}= \sqrt{(k_B T m /(\Delta t \gamma ))}~\eta (t)$, where *m* denotes particle mass, $\gamma$ a damping constant and $\Delta t$ the unit time step. Direction and magnitude of the force is randomized by the random force $\eta (t)$ chosen from a uniform distribution so that $<|\eta (t)|>=1/2$, $<\eta (t)>=0$ and $<\eta (t)\eta (t')>=2\delta (t-t')$. The Velocity-Verlet time integration algorithm was employed for updating of positions. The simulation ran for $5\times 10^7$ time-steps of $5\times 10^{-3}$. Particle positions were captured every 10,000 time-steps.

## Results

We first present observations of vesicles pool formation by optical microscopy. This is instructive on its own, but also serves for validation of the preparation, before high resolution methods are applied, such as cryo-EM or SAXS. We then present the cryo-EM results which give insights into the detailed cluster geometries of aggregated vesicles, including the adhesion zone formed by interaction of the vesicular membrane and synapsin. Finally, we show that a simple MD model can effectively reproduce the experimental observation of a finite condensate size.

### Fluorescence microscopy

Even without lipids, synapsin forms droplets above a critical concentration, see the corresponding phase diagram as a function of synapsin $c_{\mathrm{{syn}}}$ and PEG concentration $c_{\mathrm{{PEG}}}$ [19]. This manifestation of LLPS is promoted by osmotic stressors such as PEG, but is also observed without PEG. The round morphology of these droplets results from surface tension between the two liquid phases—synapsin rich and synapsin poor, and thus corresponds well to the expected phase morphology for LLPS. While results for lipid-free droplets are included in supplementary material Fig. S1, we here focus on the morphological changes and phase properties when lipid vesicles are added. All data have been recorded without osmotic stressor PEG.

**Fig. 2:**
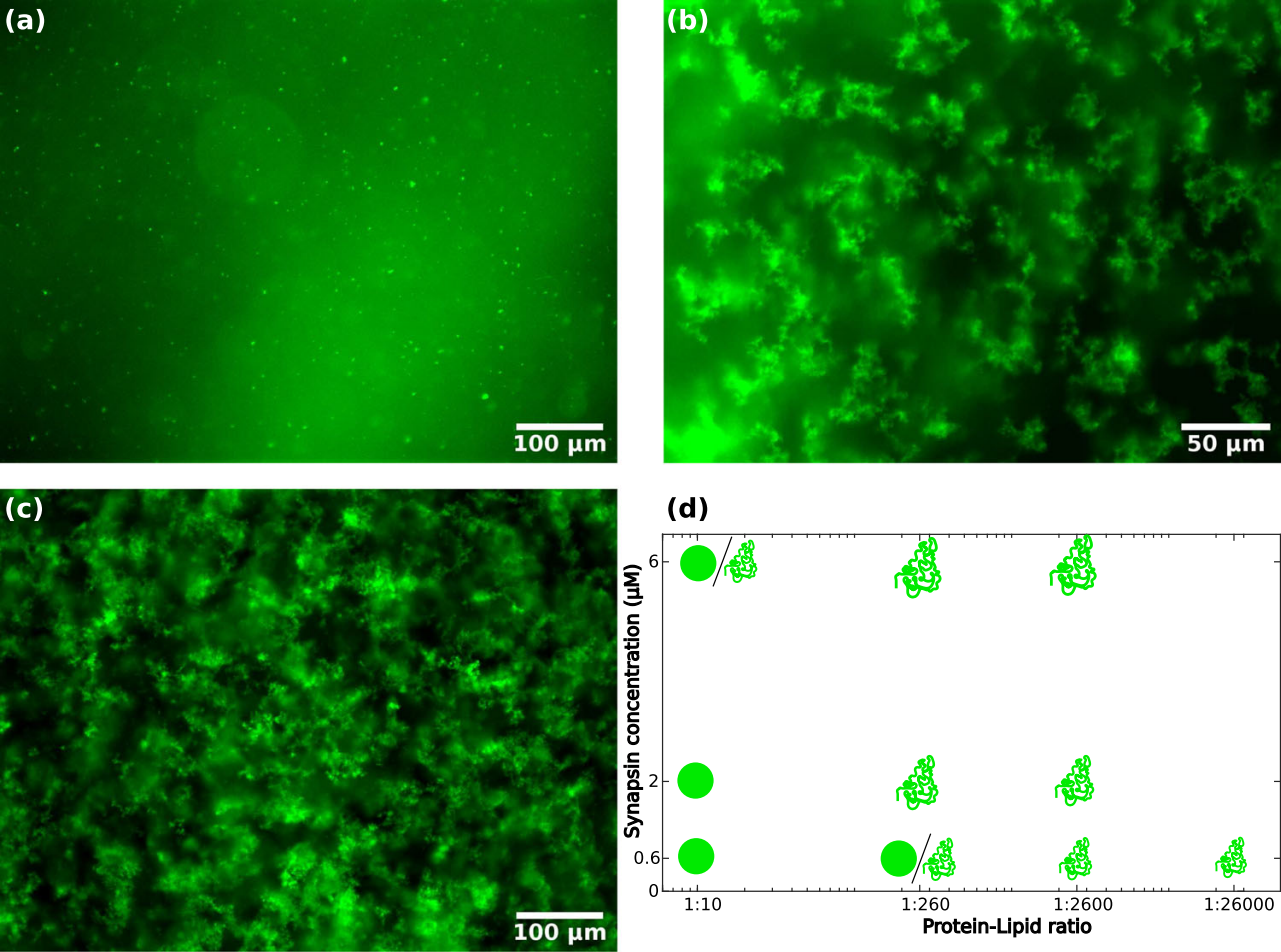
Fluorescence microscopy images of LV4-synapsin condensates for different protein-to-lipid ratios and a phase diagram for the condensate formation. **a** Fluorescence microscopy image of condensates consisting of $6\,\upmu \textrm{M}$ synapsin and $0.06\,\textrm{mM}$ LV4 ($P/L=1:10$) with a magnification of 20$\times$. **b** Fluorescence microscopy image of condensates consisting of $6\,\upmu \textrm{M}$ synapsin and $1.56\,\textrm{mM}$ LV4 ($P/L=1:260$) with a magnification of 40$\times$. **c** Fluorescence microscopy image of condensates consisting of $6\,\upmu \textrm{M}$ synapsin and $15.6\,\textrm{mM}$ LV4 ($P/L=1:2600$) with a magnification of 20$\times$. All images were taken 20–30 min after mixing. **d** Phase diagram for the formation of condensates consisting of LV4 and synapsin. Spheres indicate small spherical fractals, while the ’fractal symbol’ indicates more fractal-like condensates. Fluorescence microscopy images for all concentrations indicated in the diagram are shown in Fig. S2 in the supplementary material. Please note, that the more diffuse fluorescent signal is caused by unfocussed isolated condensates and does not stem from connections between clusters

Fig. 2a–c presents fluorescence micrographs showing the formation of vesicle condensates for LV4 and synapsin for different *P*/*L*. For (a) high $P/L=1:10$, small, spherical condensates form. For (b) lower $P/L=1:260$, the condensates are significantly larger and the morphology changes from round to fractal-like condensates, the condensate size further increases for even lower *P*/*L*, see (c) at $P/L=1:2600$. Figure 2d shows a tentative phase diagram for the morphology of condensates for different *P*/*L* and different concentrations of synapsin. Circles indicate the observation of small, spherical condensates in fluorescence micrographs, while the ’fractal symbol’ indicates the formation of fractal-like condensates. For concentrations where both symbols are shown, small fractal-like condensates as well as spherical condensates were observed. The condensate size was found to increase both with synapsin concentration and decreasing *P*/*L*, as represented by the symbol size in the diagram. With decreasing *P*/*L*, the condensate morphology bore more fractal. For completeness, fluorescence micrographs for all measures are included in the supplement, see Fig. S2.

**Fig. 3:**
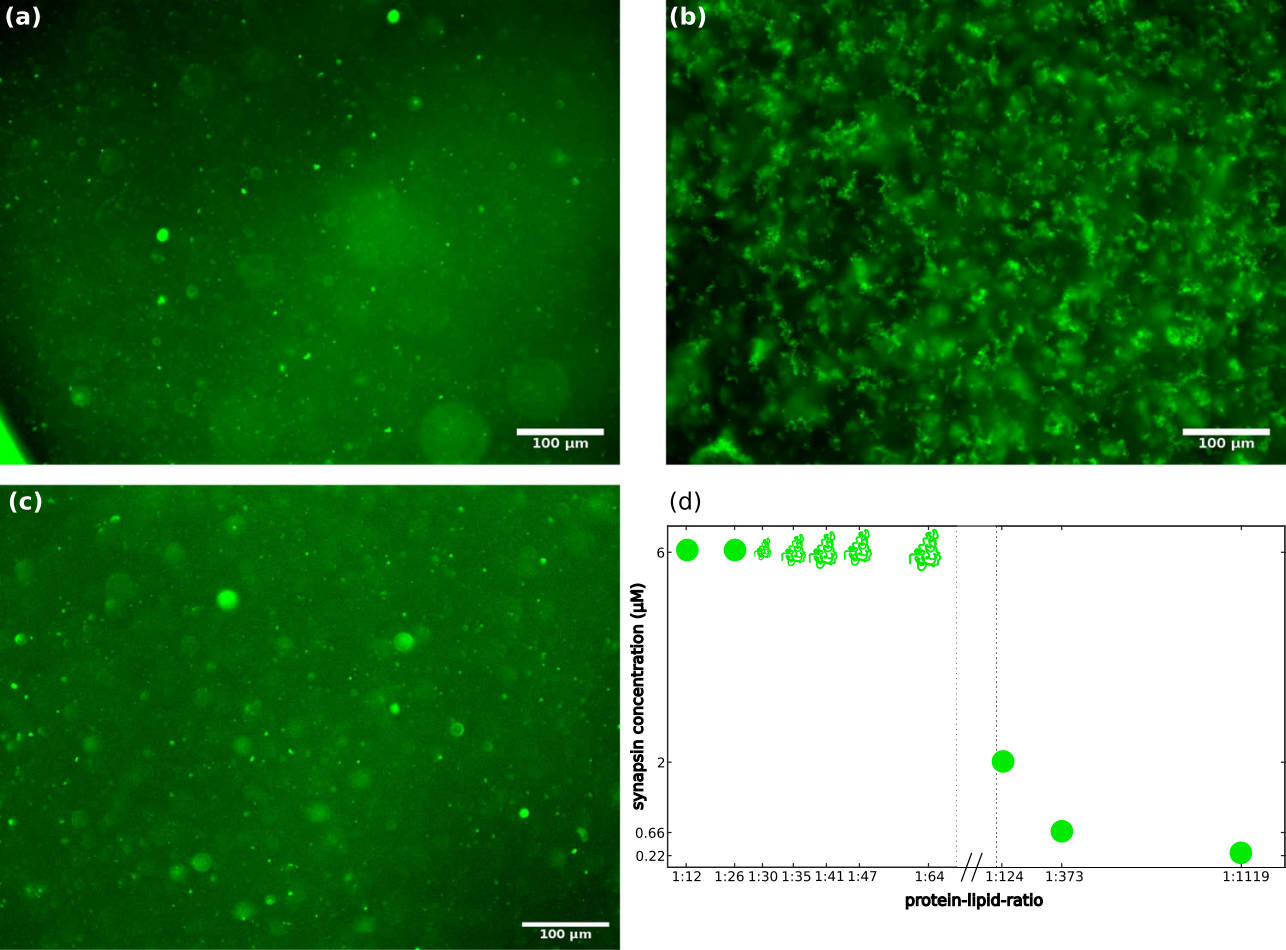
Fluorescence microscopy images of SV-synapsin condensates for different protein-to-lipid ratios and a phase diagram for the condensate formation of synapsin and SV. **a** Fluorescence microscopy image of condensates consisting of $6\,\upmu \textrm{M}$ synapsin and $10\,\textrm{nM}$ SVs ($P/L=1:12$). Small spherical condensates are visible. **b** Fluorescence microscopy image of condensates consisting of $6\,\upmu \textrm{M}$ synapsin and $35\,\textrm{nM}$ SVs ($P/L=1:41$). The lower *P*/*L* leads to the formation of fractal-like condensates. **c** Fluorescence microscopy image of condensates consisting of $0.66\,\upmu \textrm{M}$ synapsin and $35\,\textrm{nM}$ SVs ($P/L=1:373$). All images were taken at a magnification of 20$\times$ and 20–30 min after mixing. **d** Phase diagram for the formation of condensates consisting of SVs and synapsin. Circles indicate the formation of spherical condensates, the ’fractal symbol’ indicates the formation of fractal condensates. Fluorescence microscopy images for all concentrations indicated in the diagram are shown in Fig. S3 in the supplementary material. Please note, that the more diffuse fluorescent signal is caused by unfocussed isolated condensates and does not stem from connections between clusters

Fig. 3a–c shows fluorescence micrographs showing the vesicle condensate formation of SVs and synapsin for different *P*/*L* and different synapsin concentrations. At (a) high $P/L=1:12$ and high synapsin concentration of $6\,\upmu \textrm{M}$ round condensates are formed. With (b) a high $P/L=1:41$, the condensate morphology changes and the condensates show a fractal-like appearance. At (c) lower $P/L=1:373$ and lower synapsin concentration of $0.66\,\upmu \textrm{M}$, round condensates are observed. Figure 3d shows a tentative (and yet incomplete) phase diagram for the formation of condensates consisting of SVs and synapsin. The somewhat unusual simultaneous variation of *P*/*L* with a jump $c_{\textrm{syn}}$ in this experiment is explained by the fact that high absolute concentrations of lipids are not available for purified SV, in contrast to LV. Fluorescence micrographs for all *P*/*L* and synapsin concentrations measured are shown in the supplementary material Fig. S3. For a high synapsin concentration of $6\,\upmu \textrm{M}$, the morphology changes from round condensates at a high *P*/*L* to fractal-like condensates with decreasing *P*/*L*. For lower concentrations of synapsin and lower *P*/*L*, round or only very small condensates are observed.

### Cryo-EM

In order to shed more light on the microscopic structure of vesicle condensates, we have performed cryo-EM on LV4-synapsin solutions, which were allowed to incubate for a certain mixing time before vitrification by plunge freezing. Droplet formation by LLPS is hence possible and the structure aimed at is a frozen-in snap shot of the native vesicle condensate in solution. Note, however, that preparing and blotting on the EM grid before the vitrification process can induce further rearrangements, in particular in view of the strong dependence on osmotic pressure and concentrations. For this reason, it is important to first work out the protocol for pure lipid vesicle suspensions, i.e., under conditions where no vesicle condensates form.

**Fig. 4:**
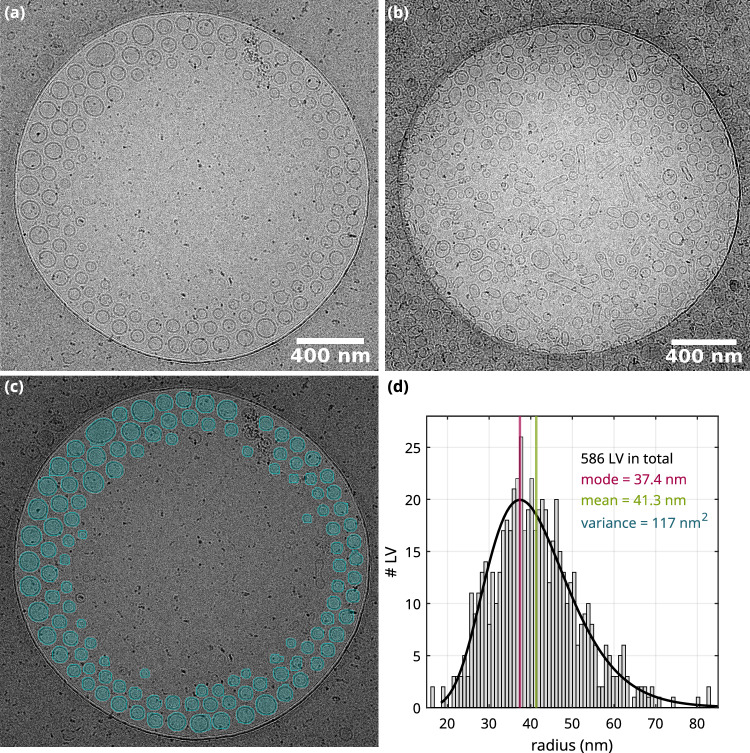
Cryo-EM micrographs of LV4 taken at a magnification of 8.7 kx. **a** LV4 (extrusion through 50 nm pore membrane, lipid concentration of 10 mM) in ultrapure water. The extrusion step in the preparation leads to an alteration of the size distribution of the vesicles. **b** LV4 (membrane pore size 50 nm, lipid concentration 15 mM) in buffer. The buffer has a significant influence on the vesicle shape. **c** Segmentation of LV4 (exemplary shown in (a)). **d** Distribution fit of LV4 radii resulting from the segmentation. The total number of segmented vesicles is 586 and the lognormal fit results in a mean of 41.3 nm with a standard deviation of 10.8 nm. It should be noted that the vesicle radii are slightly larger because the outer edge of the vesicles was not detected during segmentation

Figure 4 shows cryo-EM results for pure lipid (LV4) vesicles in both ultrapure water and buffer, along with the corresponding segmentation and quantification of the size histogram. Pure LV4 (extrusion membrane pore size of 50 nm) in ultrapure water are unilamellar, see Fig. 4a. The peculiar arrangement of vesicles sorted according to the size within the carbon film hole of the TEM grid is caused by an ice thickness gradient toward the holes center. Thus, vesicles with a smaller diameter accumulate in the middle of the amorphous ice layer. LV4 in buffer is shown in Fig. 4b. The predominantly unilamellar vesicles show a wide variety of shapes ranging from circular to highly deformed. This is in contrast to the preparations in ultrapure water which resulted in circular, unilamellar vesicles, see above. This indicates that buffer salts affect the shapes of lipid vesicles, possibly by osmotic effects, but they may also affect the formation of vesicles in the preparation by freeze-thawing and extrusion. Next, we have quantified the vesicle size distribution, which is of interest in order to characterize the preparation and in particular to understand to which extent the pore size of the extrusion filter affects the size histogram, or in other words how polydisperse the vesicles are. This requires image segmentation, which was most suitable for the preparations of LV4 in water because of the clean background, comparatively high contrast, and the well-defined spherical shapes.

To this end, individual vesicles were first segmented using the Arivis processing software [4], see Fig. 4c and further examples in supplementary Fig. S4. Segmentation was performed by first processing the raw image with Gaussian filters, followed by conversion into a binary image. Shape detection and morphological operations were then used to segment the vesicles individually (blue overlay in Fig. 4c. Almost all vesicles were detected except for a few small ones. However, the vesicle radii were systematically smaller, by about 5 nm compared to what was measured by hand. This is explained by the fact that the low contrast surfaces of the LV4 containing the lipid head groups were not completely covered by the segmentation. With this correction, the vesicle areas resulting from the segmentation were then used to compute the histogram of vesicle radii. In total, 586 LV4 vesicles were segmented from five cryo-EM micrographs (see also suppl. Fig. S4). Most vesicles exhibit radii in the range of 30 nm to 50 nm and the mean radius is $R_\textrm{mean} = 41.3\,\textrm{nm}$, as indicated in the histogram shown in Fig. 4d. It is apparent from the histogram that the radii still vary significantly resulting in a rather broad size distribution. This is not surprising given the fact that the nominal pore size of the membrane (here 50 nm) only refers to the mean of a presumingly wider distribution. Furthermore, small vesicles may pass the membrane unaffected by the extrusion cut-off associated with a pore size. Finally, the cut-off can be expected to be rather smeared out, since the vesicles can deform. It can be concluded that extrusion results in a rather broad size distribution.

**Fig. 5:**
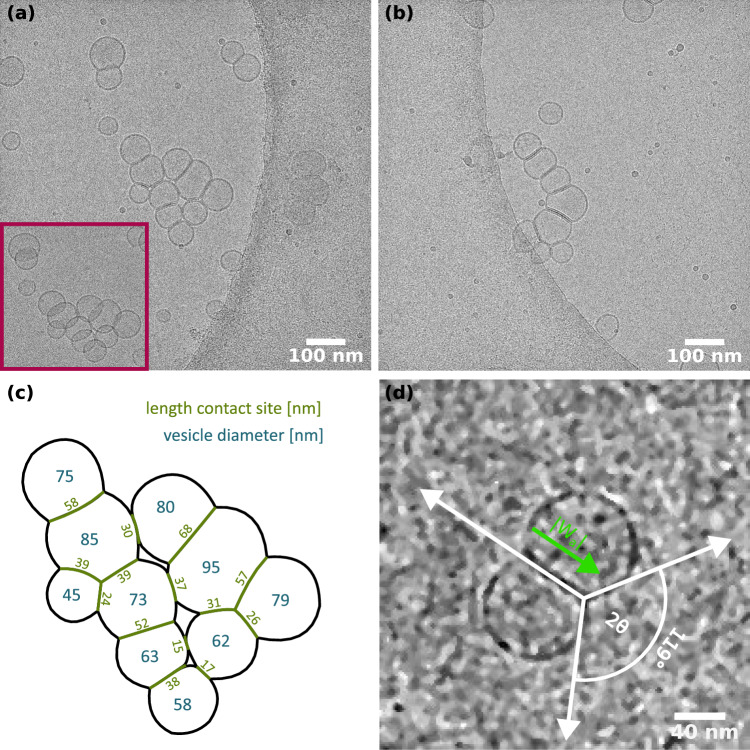
Conformational changes of the vesicles induced by the interaction with synapsin. **a**, **b**, and **d** Cryo-EM micrographs of LV4 (pore membrane size 50 nm, lipid concentration 1.56 mM) incubated with synapsin I (6 $\upmu$M) resulting in a *P*/*L* of 1:260, acquired at a magnification of 53 kx. **a** and **b** show vesicle pools. The inset highlighted in magenta is taken from the same tilt series and shows the pool from a different angle revealing the contact areas of the vesicles. **c** Contact site lengths (green) and vesicle diameters (blue) for the pool shown in (**a**). The quantities were determined manually from the cryo-EM micrograph. **d** Zoom-in of two adhering vesicles induced by synapsin. The inter-membrane interaction energy is calculated via relation (6) and results in a value of $|W_\textrm{A}|=2.83\,\mathrm {k_bT/nm^2}$ for an angle of $2\theta = 119^\circ$

With the results obtained with isolated vesicles at hand, we can now turn to the cryo-EM images of the LV4-synapsin condensates, which are presented in Fig. 5. The goal here was to resolve the inner structure of the vesicle condensates observed before by fluorescence microscopy (Sect. 3.1). For example as shown earlier, incubation of 1.56 mM LV4 and 6  $\mathrm {\mu }$M synapsin ($P/L = 1:260$) resulted in the formation of large mesoscopic domains with fractal-like morphology (Fig. 2b). Using cryo-EM, we were able to observe vesicle condensates at high magnifications (53kx, see Fig. 5a, b). Strikingly, only rather small condensates of aggregated vesicles are visible in the cryo-EM micrographs, suggesting that the larger domains are composed of smaller condensates and may have dissociated during the vitrification process. However, we can now observe the direct structural hallmark of synapsin-induced condensation, notably the pronounced adhesion areas with flattened bilayer patches. A tilt series was recorded for the particular condensate shown in Fig. 5a, as the adhesion areas become better visible when inspected at different angles (see magenta inset in Fig. 5a). We stress again that without synapsin, no such adhesion areas were observed, see the results for pure LV4 in buffer above (Fig. 4). A further control experiment with lipid vesicles consisting only of DOPC (Fig. S6) showed that there was an accumulation of vesicles induced by synapsin, but no adhesion surfaces were formed. This is an indication that anionic lipids in the vesicles are necessary for the formation of condensates. For further quantification, two parameters were determined manually from the cryo-EM micrograph: the diameter of the adhesion zone and the diameter of the vesicle, see Fig. 5c. The vesicle diameters (indicated in blue) range from 45 to 95 nm, while the cross section of the adhesion zones (green) vary from 15 to 68 nm depending on the vesicle diameter.

Next, we estimate the adhesion energy per unit area $W_a$ driving the formation of the flattened bilayer adhesion zones, i.e., the attractive contact potential of an interface which we envision as a tight layered bilayer-synapsin-bilayer system. To this end, we make use of the measured vesicle geometries and interpret them as a balance between gain in adhesion energy and elastic forces due to membrane stretching which is required to form the extra area. According to [21], the total energy *U* can be decomposed into elastic stretching energy and adhesion energy as

5 $$\begin{aligned} U=K_\textrm{a}\frac{(A-A_\textrm{0})^2}{A_\textrm{0}}-A_\textrm{f}|W_\textrm{a}| ~, \end{aligned}$$

where $K_\textrm{a}$ denotes the stretching modulus, $A_\textrm{0}=4\pi R_\textrm{0}^2$ denotes the surface area of the undeformed vesicle, and $A=A_\textrm{c}+A_\textrm{f}$ the total area of the adhering vesicle, comprising both curved area $A_\textrm{c}$ and flat adhesion area $A_\textrm{f}$. Note that in this strong-adhesion limit, curvature energy can be neglected [21]. With equilibrium contact angle $\theta$ (see Fig. 5d), we have $A_\textrm{f}=\pi R^2 \sin ^2\theta$, and $A_\textrm{c}=2\pi R^2(1+\cos \theta )$. Accordingly, the volume of the undeformed vesicle is given by $V_\textrm{0}=4/3 \pi R_\textrm{0}^3$, and the volume of the deformed vesicle by $V=\pi R^3/3 (1+\cos \theta )^2)(2-\cos \theta ))$ [21]. Furthermore, a few assumptions regarding the geometry are made. The spherical vesicles are assumed to be nearly of the same size and the volume of the vesicle lumen is set constant, as may be justified from osmotic pressure arguments. Assuming $V=V_\textrm{0}$, the radius *R* of the deformed vesicle can be expressed in terms of the contact angle $\theta$ as well as the total energy *U*. After minimizing Eq. (5) with respect to $\theta$ and some rearrangement [21], the adhesion energy per unit area can be expressed as

6 $$\begin{aligned} |W_\textrm{a}|= & {} 2K_\textrm{a}(1-\cos \theta )\nonumber \\ {}{} & {} \left[ \frac{3-\cos \theta }{(2(1+\cos \theta )^{1/2}\cdot (2-\cos \theta ))^{3/2} }-1 \right] ~. \end{aligned}$$

Using this expression and the associated approximations, we have computed the adhesion energy for three cryo-EM micrographs of adhering vesicles, one of which is shown in Fig. 5d, and the other two in supplementary Fig. S5. All micrographs were taken from the LV4 synapsin sample shown in Fig. 5a, b. Since LV4 consists of more than 50 % DOPC lipids, we took the DOPC literature value for the stretching modulus, notably $K_\textrm{a}=243\,\mathrm {mN/m}$ [22]. The adhesion energies resulting from the three micrographs and the determined contact angles are listed in Table 1. Obviously, the adhesion energy increases with a larger contact angle. At least for the two vesicles shown in Fig. 5d, the assumption of equal radii is well justified. The other two micrographs (see Fig. S5) vary slightly in diameter, which may also account for the corresponding differences in the adhesion energy.

**Table 1:** List of adhesion energies $|W_\textrm{a}|$ calculated from the equilibrium contact angle $\theta$ as well as the radii of both vesicles 1 and 2

$\mathrm {R_1}$ (nm)	$\mathrm {R_2}$ (nm)	$2\theta$ ($^\circ$)	$|W_\textrm{a}|$ ($\mathrm {k_b T/nm^2}$)
38.5	40	119	2.83
30.3	43.89	125.1	3.66
29.1	33.04	97.72	0.99

**Fig. 6:**
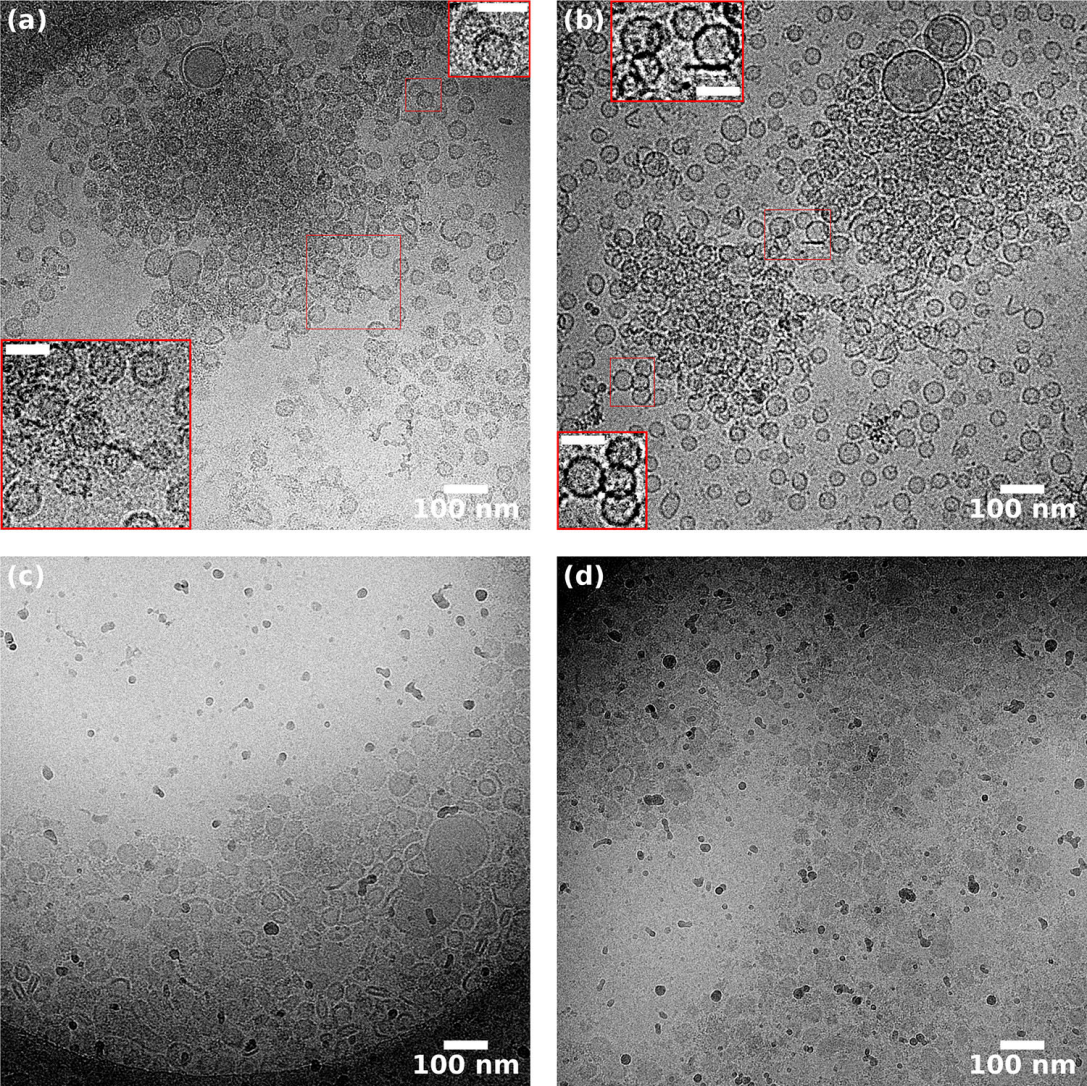
Cryo-EM micrographs of SVs (23 nM) in the presence (**a**, **b**) or the absence (**c**, **d**) of synapsin I (6 $\upmu$M, $P/L=1:27$). The images were acquired at a magnification of 42 kx. **a** A $\sim 700$ nm diameter SV condensate is surrounded by a less dense region of SVs. Individual vesicles are not discernible in the middle of the condensate, indicating that the condensate consists of multiple dense layers of SVs extending along the direction of the electron beam. Only in a few cases, tight adhesion zones are observed (see magnified insets, scale bar: 50 nm), but without visible deformations. **b** Two SV condensates of $\sim$ 600–700 nm in diameter separated by a less dense region of SVs. Importantly, most of the SVs remain spherical in the presence of 6 $\upmu$M synapsin. Some contact sites can be seen (insets, scale bar: 50 nm), but these are not flat as in the LV4-synapsin condensates (Fig 5). **c** In the absence of synapsin, most of the SVs are located on the carbon film of the TEM grid or at the edges of the holes. Many SVs are squeezed together or deformed likely due to the thin ice layer in the middle of the hole, resulting from the blotting of the sample. **d** In some cases, the ice layer was thick enough to observe SVs in the middle of the holes, in which case they tended to be less deformed than the ones in (**c**)

**Fig. 7:**
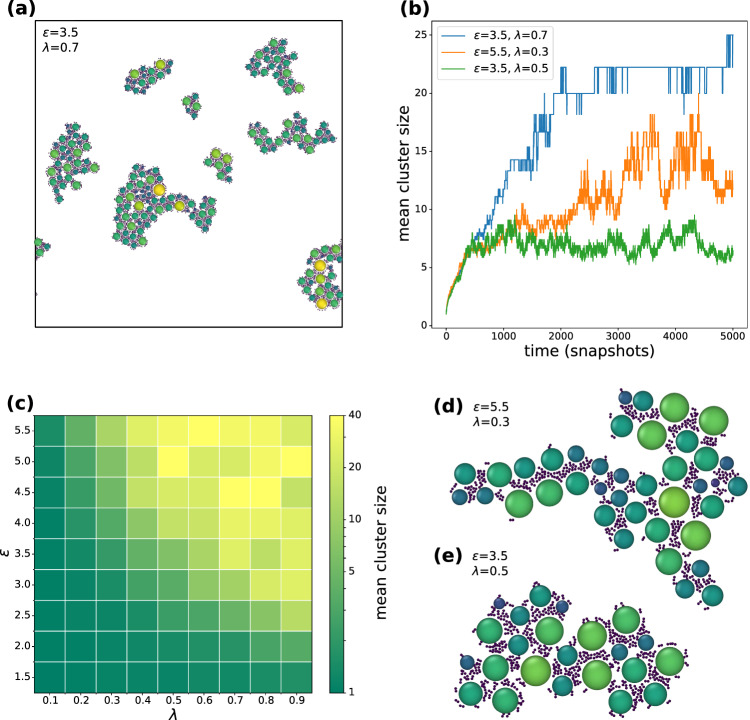
Results of molecular dynamics simulations using the bi-domain synapsin model. **a** Clusters for the final state of simulation with the bi-domain model. The attraction strength was set to $\varepsilon =3.5$, the linear synapsin density $\lambda =0.7$. **b** Growth curves for vesicle clusters. The mean cluster size is plotted over time for the bi-domain model for different attraction strengths $\varepsilon$ and linear synapsin densities $\lambda$. For $\varepsilon = 3.5$ and $\lambda = 0.5$ (green), the mean cluster size saturates. Additional growth curves are shown in supplemental Fig. S9. **c** Mean cluster size in the final state of simulation depending on the attraction strength $\varepsilon$ and the synapsin linker particles, controlled by the line density $\lambda$ in Eq. 3. **d**, **e** Examples for typical clusters in the final step of simulation for the bi-domain model with different attraction strengths and linear synapsin densities. For higher attraction strengths and smaller linear synapsin densities the systems seems to form more fractal-like structures

Next, we have also acquired cryo-EM images of the SV-synapsin system. However, since the preparation and data coverage was not as comprehensive as for the LV-synapsin sample, the results have to be considered with more caution. SV imaging is also more challenging, since many more impurities and membraneous fractions are often observed, and it is often unclear whether this is associated with the collection and purification protocol, or whether this is further aggravated by vitrification.

Figure 6 presents a selection of cryo-EM micrographs of SVs (23 nM) with (a,b) or without (c,d) synapsin I (6 $\upmu$M, $P/L=1:27$), acquired at a magnification of 42kx. In (a) and (b) condensates are observed, surrounded by less dense regions of dispersed SVs. Individual vesicles are not discernible in the middle of the condensate, indicating that the condensate consists of multiple dense layers of SVs extending along the direction of the electron beam. The SVs at the periphery of the condensate are slightly separated from their neighbors and only in a few cases can we see tight adhesion zones, but without the deformations seen as for the LVs above. Indeed, most of the SVs exhibit spherical shape as in pure SV images without synapsin. The contact sites are small compared to the size of the SVs, as opposed to the extended adhesion zones observed in the LV4-synapsin condensates. In the absence of synapsin, most of the SVs are located on the carbon film of the TEM grid or at the edges of the holes, see (c,d). Many SVs are squeezed together or deformed, but in this case, this is likely due to the thin ice layer in the middle of the hole, resulting from the blotting of the sample. Note that the lower viscosity of SV in buffer may result in a much thinner ice than for SV-synapsin. In some cases, the layer was thick enough to observe SVs in the middle of the holes, as for example in (d). They tended to be less deformed than the ones in (c). In pure SV samples, we did not observe the kind of condensates as for the SV-synapsin, see (a) and (b). Nevertheless, the SV distribution was rather inhomogenous, with regions of variable density making it difficult to distinguish between true condensates and artifacts arising from sample vitrification.

### Molecular dynamics simulations

The primary objective of the molecular dynamics (MD) simulations was to investigate the growth and saturation of condensates, comparing simplified models to experimental observations. Notably, we wanted to conceive and explore minimal models capturing the dynamics of vesicle condensation and condensate formation, including the observed saturation of condensate growth. To avoid numerical complexity and to exploit the performance of existing simulation packages, notably the LAMMPS simulation software, we focused on two dimensional (2*d*) simulations and all interacting particles were treated as round objects with pairwise interaction forces, as detailed in Sect. 2.4. Each vesicle was decorated with synapsin molecules with a constant surface density $\lambda$. Each synapsin molecule was bound to the vesicle by a harmonic spring potential, but it could move freely along the surface.

Two different formulations were tried out, treating synapsin either as a single particle (mono-domain model) or as two particles (bi-domain model) with correspondingly more complex set of interactions, differing for the two beads. To define, which vesicles belong to the same cluster, an Axis-Aligned Bounding Box (AABB) tree [13] was employed. Identifying clusters solely based on vesicle position proved unreliable because vesicles could be in close proximity without being connected by synapsin. For this reason, we first determined which synapsins interacted with each other. Synapsins closer than 4 ($8\times r_{\mathrm{{vesicle}}}$) were assigned to the same cluster. Subsequently vesicles were assigned to a cluster if their synapsins belonged to the same cluster.

In the mono-domain model, synapsin is described by a single particle with fixed radius, while vesicle particles are taken from a distribution. The model led to the fusion of synapsin from one individual vesicle into a single cluster with almost no distance between the synapsin particles. As a consequence, no synapsin is available for interaction with synapsin of another vesicle and further cluster growth is prevented. While this evidently reproduces growth saturation (Fig. S8 in the supplementary material), the cluster size with an average of three vesicles per cluster is clearly unrealistically small. To overcome this limitation, a second model, the so-called bi-domain model was conceived, in which synapsin is described by two round particles.

Figure 7 shows the results for this bi-domain synapsin model, with (a) presenting a snapshot at the final state of simulation. Exemplary growth curves for different values of the attraction strength $\varepsilon$ and the linear synapsin density $\lambda$ are shown in (b). Additional curves are depicted in the supplemental Fig. S9. The mean cluster sizes are presented in a two-dimensional plot as a function of interaction strength $\varepsilon$ and synapsin density $\lambda$ in Fig. 7c. For small $\varepsilon$ and/ or small $\lambda$, no clusters a formed. At intermediate $\varepsilon$ and $\lambda$, clusters form but saturation of the cluster size is observed (green). In this regime, the mean cluster size is much smaller then the total number of particles in the system. For higher $\epsilon$ and $\lambda$ (orange and blue), the clusters grow continuously over the entire simulation run of 5000 time units (so-called snapshot), with one time unit corresponding to $10^{4}$ MD steps. This is accompanied by larger mean cluster sizes. The fluctuations in the growth curves are due to fusion and breaking of single clusters. Representative clusters for two combinations of $\varepsilon$ and $\lambda$ are shown in (d) and (e).

## Discussion, conclusions and outlook

As we saw above, fluorescence light microscopy is well suited for imaging the mesoscale morphology of the vesicle aggregates induced by synapsin. The synapsin and lipid rich domains which are not always found to be of a spherical shape, but exhibit a transition from round domains to larger almost percolating aggregates with fractal appearance at high synapsin concentration $c_{\mathrm{{syn}}}$ and lower lipid-to-protein ratio (small *P*/*L*). A simple view of liquid–liquid phase separation (LLPS) would let us expect rather round interfaces governed by interfacial tension between two fluid phases. In the present case, however, only one phase would correspond to an ordinary fluid, while the other would be a structured fluid engulfing the vesicle pools, a kind of meta fluid. If vesicle pool formation by synapsin is regarded as a manifestation of LLPS, the LLPS scenario must hence be generalized or widened to some extent, including also the possibility of more complex condensate shapes and shape transitions. In fact, shape transitions in LLPS are still debated [28] and have been discussed also in view of a liquid-to-solid transition [1]. However, the question of condensate shape has to be distinguished from the question of the phase state, i.e., whether a condensate is fluid or solid. For the latter, criteria of shear viscosity and/or diffusion constant seem better suited than the shape of the phase boundary. Interestingly, complex shapes also occur in other biological condensates than the present vesicle-synapsin system, notably in condensates formed by amyloid proteins [20]. Finally, we note that shape transition have also been observed in lattice gas models [35].

Aside shape transitions, the growth scenario and self-limiting growth is an important aspect of LLPS. Again, a simple-minded view would predict coarsening of domains and domain coalescence, by spinodal decomposition or nucleation and growth. For round droplets it is sometimes difficult to clearly tell apart a slowing down of phase separation kinetics from true saturation of growth. This is because as the domains mature and the diffusion constant decreases, the times between two phases getting into contact increases. In the present case, however, we believe that the latter (saturation) better accounts for the observations. This is particularly evident for the fractal-like condensate shapes, which at high concentration almost seem to percolate such that contact probability does not seem to be a limiting factor. Importantly, in this case as well no further compactification with time is observed, indicating that despite attractive forces between vesicles and synapsin, the lowest energy state does not correspond to a compact ’complex fluid’ of vesicles and synapsin.

For this reason, the question arises which effects lead to growth saturation. Theoretically, a finite length scale for the condensates must derive from the balance between short range attractive forces between vesicles in the presence of synapsin and repulsive long range interactions. Since electrostatic interactions are screened out and cannot account for the long range repulsion, we speculated in the introduction that elastic forces effectively cut-off further growth of condensate size. With the cryo-EM data at hand, we have now obtained insight into the nanoscale vesicle structure and can put forward a mechanistic explanation of elastic stress of vesicle membranes may lead to growth saturation. In fact, we found that the addition of synapsin to LV4 results in the formation of flat adhesion zones, hence a deformation which must be associated with stretching of the bilayer in order to accommodate the area increase. This elastic energy may then limit the number of other vesicles which can bind to a given vesicle. In other words, vesicles which are pre-stretched from previous adhesion sites may not be able to accommodate more neighboring vesicles. As the condensate grows, the surface of the condensate becomes increasingly stressed, with fewer possible contact sites, inhibiting further growth. The driving force for this is the high interfacial energy ranging from $W_\textrm{a}= 1 \,\mathrm {k_BT /nm^2}$ to $W_\textrm{a}= 4\,\mathrm {k_BT /nm^2}$, which is released when the bilayers flatten to achieve close apposition with each other mediated by synapsin in the layered adhesion zone. Interestingly, this synapsin-mediated contact was only observed for anionic lipids, as the control experiment with pure DOPC (and no DOPS) showed neither contact sites in the cryo-EM images, nor any droplets or other forms of aggregation. This points to the fact that the synapsin-bilayer interaction is governed by strong electrostatics (similar to $CaCl_2$ induced adhesion [14]), and at least not exclusively governed by hydrophobic interactions. Next, we tried to build upon the key idea that elastic stress following adhesion induced membrane extension and strain limits the size of the condensates. In essence, even if synapsin was present in sufficient concentration, i.e., in excess, the available adhesion area depletes when a vesicle builds up successive contact sites. This will affect both geometry of vesicle packing as well as the overall size. To capture the essential (minimal) physics of this idea, we have formulated a simple molecular dynamics model, using only (effective) spherical particles with suitably defined interactions. In this model, the synapsin particles represent bound synapsin to adhesion sites with stretched membranes, and the fixed concentration of synapsin particles per vesicle accounts for the limited adhesion area due to membrane elasticity. As two medium size clusters have formed, they will both be saturated which means they will no longer attract each other as soon as they both have gained a certain size. As the simulation showed, this size limit can indeed be observed for a reasonable set of parameters. Certainly, the particular choice of the model can be argued and is far from unique. Further much more involved simulations could extend this work to three dimensions and could also explicitly including vesicle deformation and membrane elasticity.

For synaptic vesicles, however, the case is entirely different. Flattened adhesion zones are clearly neither functionally reasonable in synaptic vesicle pools, nor are they observed in TEM images of synapses. This can clearly be ruled out. In this work or reconstituted cell-free model systems, we also included first images of purified SVs and synapsin (SV-synapsin), for comparison with the LV-synapsin system, which was the main focus of the cryo-EM investigations. Indeed, no flattened adhesion zones or any tight contacts with membrane deformations were observed. At the same time, at least in some images, the capability of synapsin to form SV pools or condensates was clearly observed, even if many SVs were still present outside these dense condensate regions. Why then are tight adhesion zones with membrane deformations not observed in SV-synapsin? It seems plausible that the dense layer of vesicle proteins impedes the assembly of high synapsin coverage which then leads to the flattening and stretching of membranes. Such a strong binding and deformation seems to requires a pure lipid bilayer. While it is more difficult to achieve convincing cryo-EM images for the SV-synapsin system in reconstituted systems, our first results on this system clearly rule out overly tight adhesion zones as in LV-synapsin. Note, however, that also in this case elastic interactions could play an important role, since the synapsin ’network’ around undeformed and morphologically intact SVs may also carry elastic energy.

To better distinguish SV condensates from vitrification induced accumulations of vesicles and to reduced impurities or ruptured vesicles, future work should include a variation of blotting parameters, and also vitrification of freshly purified SVs without intermediate freezing for storage of SV preparations. Complementary high resolution techniques such as super-resolution fluorescence microscopy by stimulated emission depletion (STED) and small-angle X-ray scattering (SAXS) should also be used for this purpose.

Furthermore, future extension of the present work should be directed at a quantification of condensate size as a function of time *t*, which, however, is not straightforward. In the present experiments, smaller condensates or droplets were below the diffraction limit, and larger condensates often sedimented to the chamber floor. By tracking of the droplet trajectories, reasonable values for the effective condensate or droplet sizes could possibly be obtained from the diffusion constants *D*, but this would only be reliable for round droplets. Super-resolution imaging or vitrification for a range of well-controlled incubation times would be a further possibility.

### Supplementary Information

Below is the link to the electronic supplementary material.

Supplementary file 1 (pdf 8377 KB)

## Data Availability

The datasets generated during and/or analyzed during the current study are not publicly available, in part also due to their significant size, but are available from the corresponding author upon reasonable request.
