# Supplementary material for: Vesicle condensation induced by synapsin: condensate size, geometry, and vesicle shape deformations
Source: Eur Phys J E Soft Matter. 2024 Jan 25;47(1):8. doi: 10.1140/epje/s10189-023-00404-5 (PMC11233366; doi:10.1140/epje/s10189-023-00404-5)
Supplement: Supplementary file 1 — (pdf 8377 KB) [file 10189_2023_404_MOESM1_ESM.pdf]

Supplementary Material to "Vesicle condensation induced by synapsin: condensate size, geometry, and vesicle shape deformations"

Jette Alfken[1†], Charlotte Neuhaus[1†], András Major[1], Alyona Taskina[1,5], Christian Hoffmann[2], Marcelo Ganzella[3], Arsen Petrovic[4], David Zwicker[5], Rubén Fernández-Busnadiego[4], Reinhard Jahn[3], Dragomir Milovanovic[2], Tim Salditt[1*]

[1*]Institut für Röntgenphysik, Georg-August-Universität, Friedrich-Hund-Platz 1, Göttingen, 37077, Germany.
[2]Molekulare Neurowissenschaften, Deutsches Zentrum für Neurodegenerative Erkrankungen (DZNE), Charitéplatz 1, Berlin, 10117, Germany.
[3]Labor für Neurobiologie, Max-Planck-Institut für multidisziplinäre Naturwissenschaften, Am Fassberg 11, Göttingen, 37077, Germany.
[4]Institut für Neuropathologie, Universitätsmedizin Göttingen, Justus-von-Liebig-Weg 11, Göttingen, 37077, Germany.
[5]Theorie biologischer Flüssigkeiten , Max-Planck-Institut für Dynamik und Selbstorganisation, Am Fassberg 11, Göttingen, 37077, Germany.

*Corresponding author(s). E-mail(s): tsaldit@gwdg.de;
Contributing authors: ;
†These authors contributed equally to this work.

1 Additional FLM and cryo-EM datasets

V1: Video of the cryo-EM tilt series of LV4.

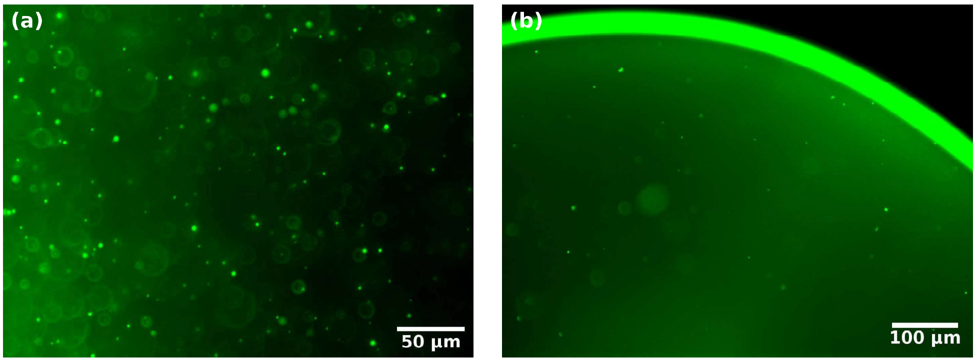

Supplementary Fig. S1 Condensate formation of synapsin without lipids at a concentration of $c_{syn} = 6\,\mu$M. (a) Fluorescence micrograph recorded 30 minutes after mixing synapsin with 3% PEG, at a magnification of 40x. Small, spherical synapsin condensates are formed. (b) Synapsin at a concentration of $c_{syn} = 6\,\mu$M, recorded 60 minutes after preparation of the solution at a magnification of 20x. A few small, spherical condensates are visible. The bright curved interface at the top of the image corresponds to the rim of the droplet which is enriched with synapsin.

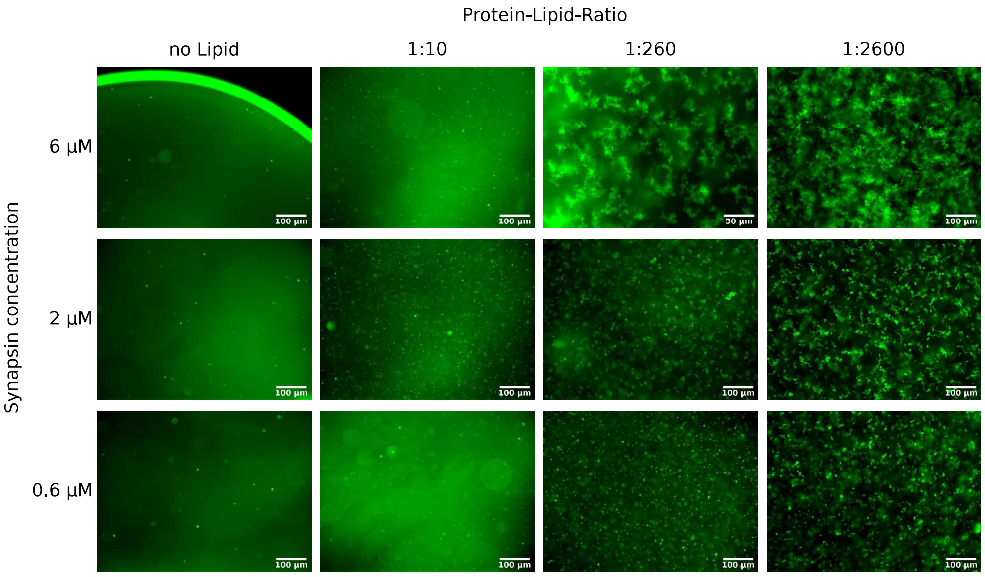

Supplementary Fig. S2 LV4-synapsin condensates for different P/L and c_{syn}. Fluorescence micrographs were acquired 20 minutes after mixing. All images were recorded at a magnification of 20x, except for the image of $P/L = 1:260$ and $c_{syn} = 6\,\mu$M, which was recorded at 40x. Please note, that the more diffuse fluorescent signal is caused by unfocussed isolated condensates and does not stem from connections between clusters.

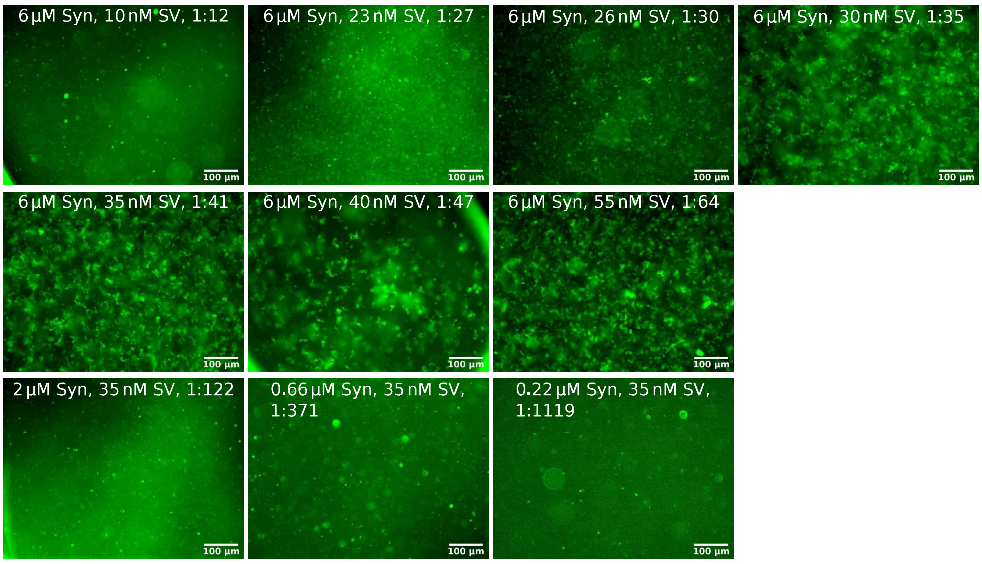

Supplementary Fig. S3 SV-synapsin condensates for different P/L and c_{syn}. The absolute synapsin (Syn) and SV concentrations and P/L are indicated in the figure. Fluorescence micrographs were recorded 20 minutes after mixing at a magnification of 20x. Please note, that the more diffuse fluorescent signal is caused by unfocussed isolated condensates and does not stem from connections between clusters.

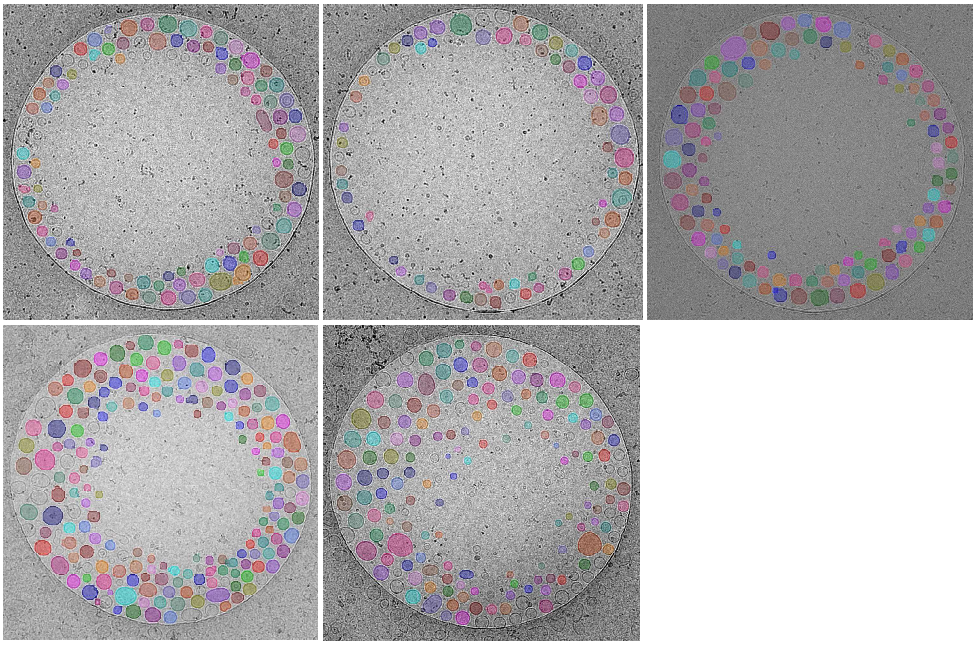

Supplementary Fig. S4 Segmentation of LV from EM-micrographs. The vesicles are slightly larger than determined by segmentation. An offset radius of 5 nm is measured.

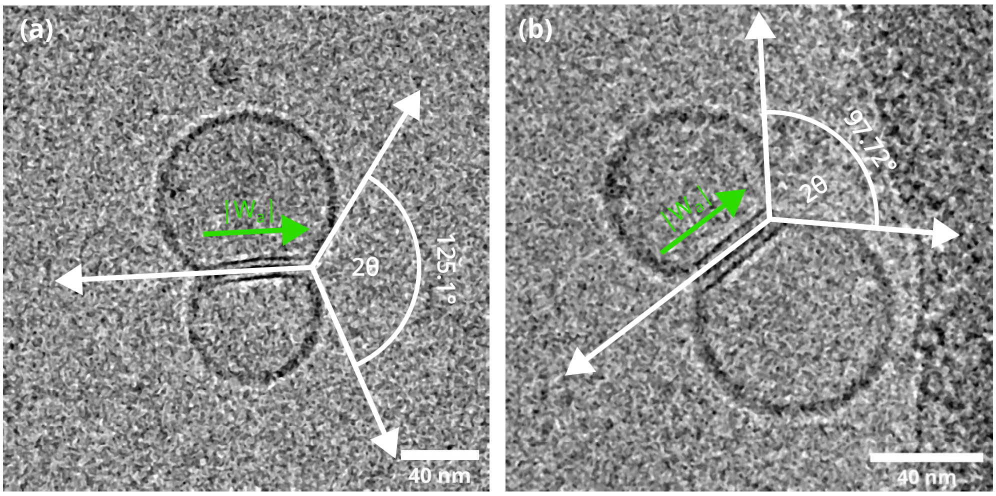

Supplementary Fig. S5 Cryo-EM micrographs of two adhering LV4 induced by synapsin binding as well as the determined contact angle 2θ.

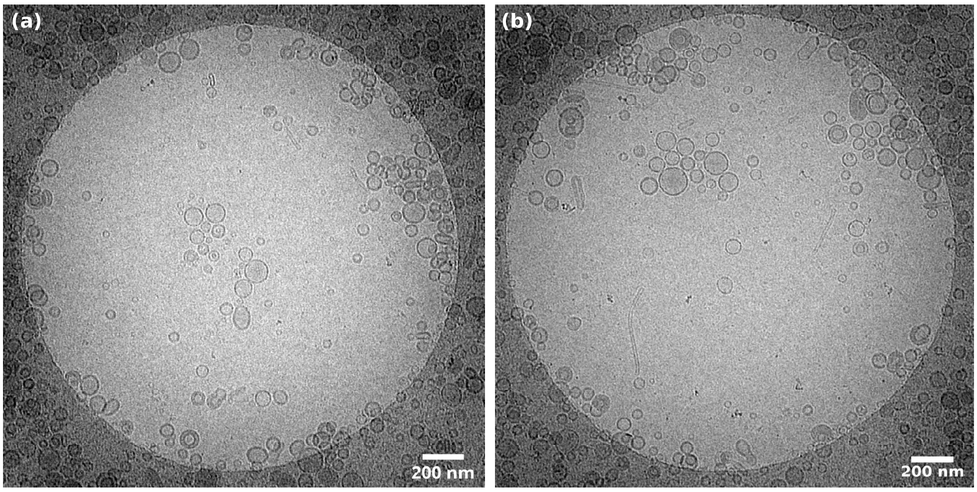

Supplementary Fig. S6 Cryo-EM micrographs of pure DOPC vesicles (lipid concentration 1.56 mM) incubated with 6 μM synapsin. These control experiments reveal an aggregation of lipid vesicles after mixing with synapsin. Strikingly, no adhesion zones are formed as in the presence of anionic lipids and synapsin.

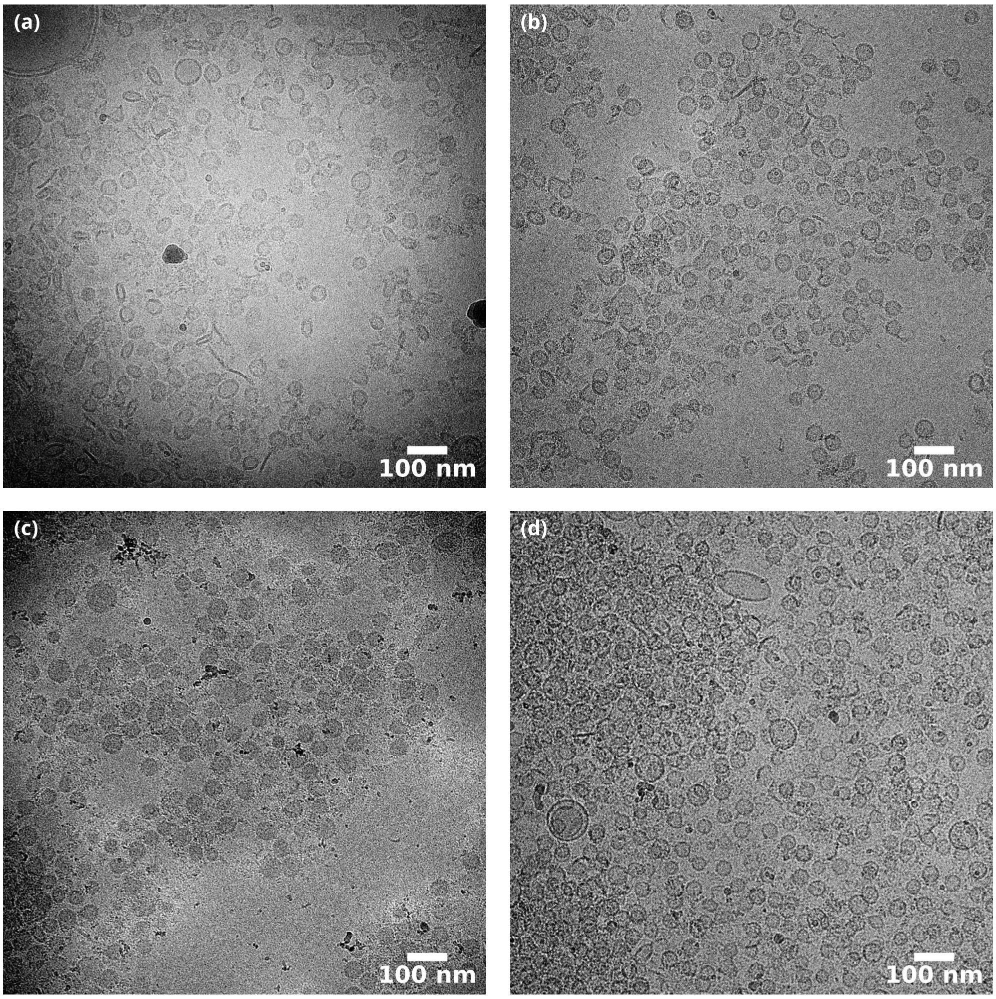

Supplementary Fig. S7 Cryo-EM micrographs of SVs (35 nM) with different concentrations of synapsin. All images were acquired at a magnification of 42kx. (a) SVs without synapsin. (b) SVs with 0.66 μM synapsin ($P/L = 1:373$). (c) SVs with 2 μM synapsin ($P/L = 1:124$). (d) SVs with 6 μM synapsin ($P/L = 1:41$).

2 MD simulation

Fig. S8 presents the results for the previously mentioned mono-domain model. A snapshot of aggregated vesicles is shown in (a). The mean cluster size for different combinations of the attraction strengths ε and linear synapsin density λ is depicted in (b), while (c) shows the increase of the vesicle pool diameter with simulation time, clearly evidencing saturation of growth. For this the AABB tree was applied for each snap-shot and the mean cluster size defined as the mean of all cluster sizes according to the AABB tree was calculated. The curves show that the cluster growth mainly occurs in the first 300 snapshots (3000000 time steps) and then plateaus at a mean cluster size of around 3 vesicles per cluster, which is lower than the fluorescence microscopy measurements. Larger ε as well as larger λ can be associated with a smaller lipid to synapsin ratio in the experimental system. For values of $\lambda > 0.15$ the mean cluster size lies between 1.5 to 3.5 vesicles per cluster and does not seem to depend on the two parameters, small λ values, e.g. $\lambda = 0.08$, result in a reduced cluster size. There is no cluster formation in the absence of synapsin ($\lambda = 0$). As mentioned in the main text, this model is limited by the clustering of synapsins of an individual vesicle, resulting in the prevention of growth.
V2-4: Videos of the selected trajectories shown in Fig. 7, $\varepsilon = 3.5$ $\lambda = 0.5$, $\varepsilon = 3.5$ $\lambda = 0.7$ and $\varepsilon = 5.5$ $\lambda = 0.3$ respectively. Every 10th snapshot is shown.

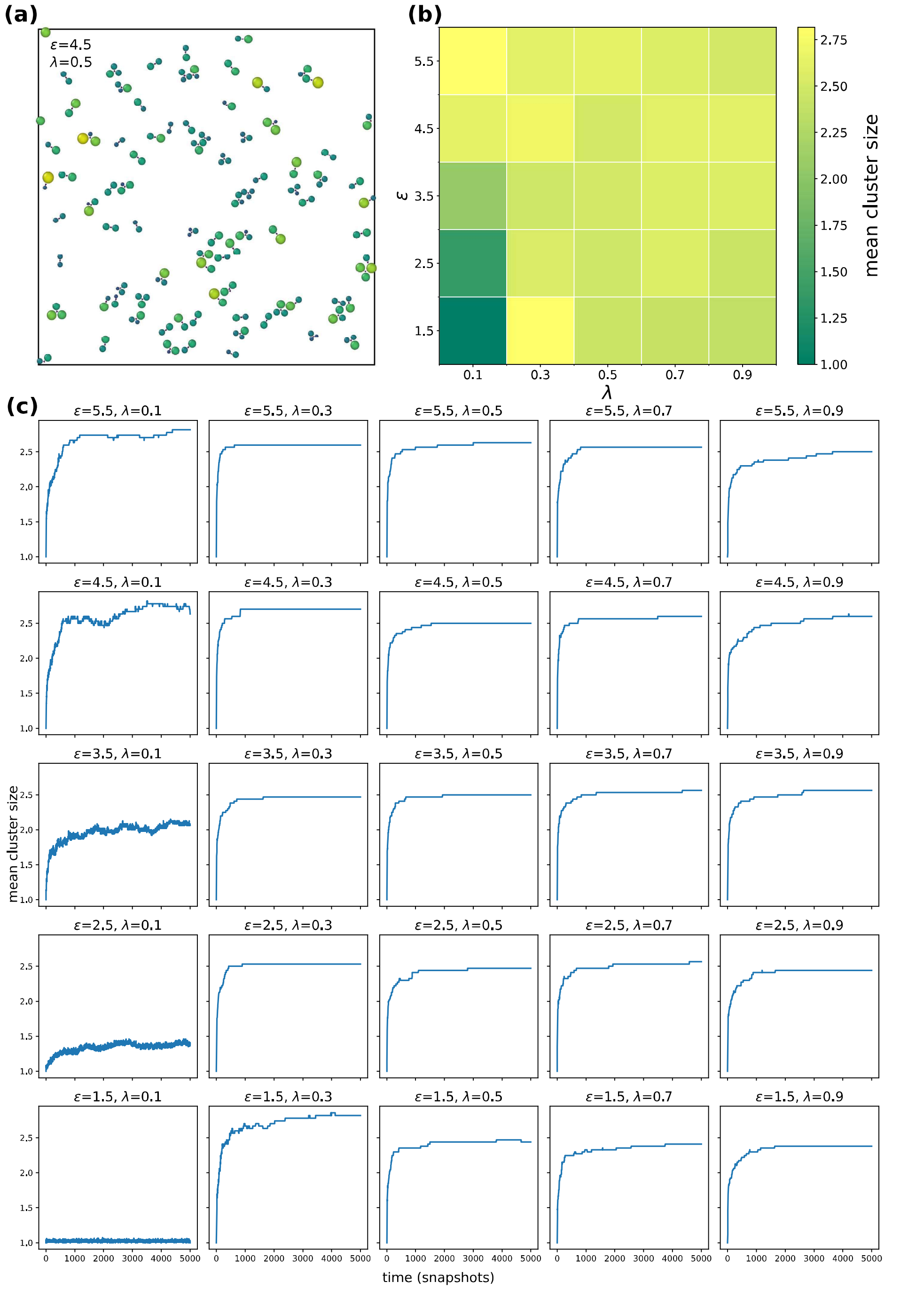

Supplementary Fig. S8 Results for molecular dynamics simulations using the mono-domain synapsin model. (a) Clusters for the final state of simulation with the mono-domain model. The attraction strength was set to $\varepsilon = 4.5$, the linear synapsin density $\lambda = 0.5$. (b) Mean cluster size in the final state of simulation depending on the attraction strength ε and the linear synapsin density λ. (c) Growth curves for different combinations of ε and λ.

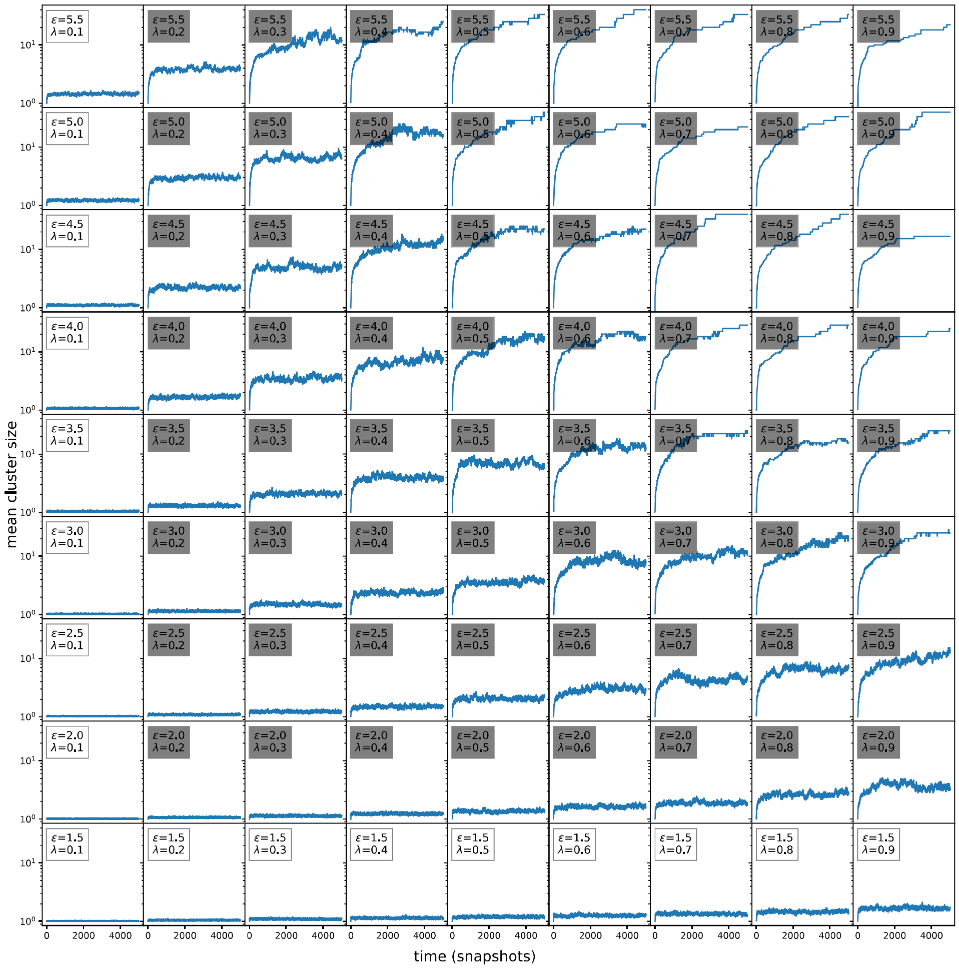

Supplementary Fig. S9 Growth curves for different combinations of attraction strength ε and linear synapsin densities λ. For small ε and/ or λ the growth curves reach a plateau. For higher values the cluster keeping growing.
